# Case of Chronic Diarrhœa, Accompanied by the Discharge of Some Peculiar Substances

**Published:** 1817-09

**Authors:** Jeremy Stimson

**Affiliations:** of Dedham.


*For the London Medical and Physical Journal.*

### *Case of Chronic Diarrhœa, accompanied by the Discharge of some peculiar Substances;* by Dr. Jeremy Stimson, of Dedham.

MARY-ANN CLAP came under my care on the 6th of May, 1816, at which time she was ten years of age. Her disease commenced in July, 1814, and had continued from that period with great uniformity of symptoms. She had had a constant diarrhœa, having had from four to six or eight dejections daily, without an intermission, for twenty-four hours. The discharges had at some times been attended with pain, but this had never been severe. Her appetite had been great during the whole period, and within a few months past it had been voracious, so that she had consumed as much food as would have served two or three healthy men. She had, notwithstanding, a faintness of the stomach, which was relieved only for a short time after eating. It appeared from her evacuations that her food was not well digested. During her sickness she had been very gradually losing flesh; and, as far as could be judged from her clothes, she had not increased in height. Her strength had not been so much impaired as might have been expected; she had not been confined, and had even attended school most of the time until within a few weeks. She had taken many simple medicines, but without benefit; and had within a week taken a powerful cathartic, which seemed to have been useful.

Such was the account which I received from her mother at my first visit. Her countenance was now pale and sickly, pulses rather feeble, and, as I judged, about ninety in a minute. Her tongue was slightly coated. Her abdomen was enlarged. Her appetite had been somewhat diminished, and the diarrhœa checked since the use of the cathartic. Influenced partly by the effect of that remedy, I directed a dose of jalap with submuriate of quicksilver, and requested the mother to examine the discharges carefully.

On May 8th I was informed that the medicine had operated powerfully, and that it had brought off a number of white substances with much dark gelatinous matter. The white substances were said to be about two-thirds of an inch in length, and, from the description, I was led to believe that they were portions of a tænia. Afterwards, when I had an opportunity of examining these substances, I found them to differ altogether from the joints of the tape-worm. They were of various forms and sizes, some not larger than a pea, others others as large as a filbert. Some of them were nearly spherical, but they were mostly in a form nearly resembling a bean. Their composition was homogeneous, and it was evident that they were not organized bodies. They were firm substances, and, when cut, they appeared to resemble tough new cheese more nearly than any thing else. I was induced to think, however, that they consisted of coagulable lymph rolled up, although they were more dense than this substance commonly is. On one of them was perceived a slight red speck. Examining this with a magnifying glass, it was found to arise from a little dot of blood, and this extended into the substance for a little distance, as if a small vessel had penetrated it, and had been broken off.

At first the disease was treated as a case of tænia. Half an ounce of oil of turpentine was given, and this was followed by a cathartic. Hundreds of the white substances were thus brought off. As the patient's strength was very little reduced by these remedies, and her symptoms rather mended, the cathartic was repeated two or three times during May and June. Under the operation of those medicines, many more of these substances were discharged, and indeed they appeared in the stools also at other times.

In consultation with Dr. Jackson of Boston, it was agreed that these peculiar substances must probably be formed in the intestines, in consequence of a morbid state of some part of the mucous membrane. Under this belief it was agreed to administer small doses of submuriate of quicksilver and tartrite of antimony with opium at night for a short period, and then to try the phosphate of iron; at the same time to allow a nutritious diet, and to enjoin daily exercise. Although my patient's strength was now reduced, she bore riding very well, and derived great pleasure from it. It was agreed that if she continued to lose strength under the course prescribed, without decided relief as to her principal complaints, the medicines should be omitted. She did lose strength under this course, her appetite diminished, and her diarrhœa increased, so that after a few days the medicines were omitted. There was also some change in her symptoms which influenced this decision; for, on the 5th of July, there supervened a violent spasmodic cough, which lasted an hour and a half, and was at last arrested by an anodyne. On the following night she had an ague-fit. The discharges on the 6th were frequent, and of a cream colour. She then took tincture of kino with tincture of opium. On the 7th she had a soreness or pressure in the epigastric and right hypochondriac regions, for which the part was vesicated. On the 8th she had violent tenesmus, which was relieved by an enema of starch with tincture of opium. From this time she failed rapidly, her diarrhœa continuing, until the 15th, when she expired.

On the morning after death I examined the cavities of the thorax and abdomen. The lungs were healthy in their appearance, but the left lobe adhered extensively to the pleura costalis. The heart was rather small, but healthy; the pericardium contained a little more fluid than usual.

On viewing the viscera of the abdomen *in situ naturali*, the following circumstances were noticed. The omentum was small, and destitute of fat. The small intestines were of their natural colour, and had their usual polish. The large intestines appeared more dark-coloured than usual, particularly certain parts. These were, the cœcum; the first portion of the colon, in extent, about five or six inches; and its latter portion, in extent, about three or four inches. In these parts the vessels seemed filled, as if by injection; but the peritoneal coat had its usual polish. The liver was larger than natural, and extended quite into the left hypochondrium. Its right lobe was thickened, and was both hard and more dark-coloured than the left lobe. The gall-bladder was full, and of its natural form: its duct, and those connected with it, were pervious. The spleen and pancreas appeared in all respects sound.

The alimentary canal was laid open through its whole extent, except the gullet. The stomach contained air with a dark-coloured fluid, not offensive in smell, and in quantity about half a pint. The small intestines contained a fluid not very dissimilar to that in the stomach, except that in the lower part of the ileum it had more consistence, and resembled dark moist clay. Through the small intestines there was not any vestige of disease, except that the valvulæ conniventes were preternaturally pale, and destitute of mucus. On laying open the large intestines, there were evident marks of disease. These were seen in the cœcum, and in the first and last portions of the colon. In these parts the mucous membrane was very red, and much swollen; the surface was rough, but did not exhibit any appearance of ulceration. The diseased portions of the intestines felt nearly as thick again as the healthy portions. The inflammation extended all around the bowel, and terminated abruptly. The large intestines contained a dark-coloured matter, similar to that which the patient had voided for some days previous to her death. One of the mesenteric glands was enlarged and indurated.

It is worthy remark that there was not found, in any

 part part of the alimentary canal, any thing resembling, in the smallest degree, the peculiar substances which the patient had discharged so abundantly during life.—*New-England Journal.*

